# TRIB3 facilitates glioblastoma progression via restraining autophagy

**DOI:** 10.18632/aging.103969

**Published:** 2020-11-16

**Authors:** Zhanbin Tang, Hongping Chen, Di Zhong, Wan Wei, Lili Liu, Qiong Duan, Baichao Han, Guozhong Li

**Affiliations:** 1 Department of Neurology, The First Affiliated Hospital of Harbin Medical University, Harbin, Heilongjiang Province, China

**Keywords:** glioblastoma, tribbles pseudokinase 3, autophagy

## Abstract

The pseudokinase Tribble 3 (TRIB3) is known as a regulator in cellular responses to a variety of stresses, such as glucose insufficiency and endoplasmic reticulum (ER) stress. TRIB3 is upregulated in various cancer tissues and is closely connected to the poor prognosis of patients. However, the underlying regulation and function of TRIB3 in glioblastoma (GBM) is still largely unknown. In this study, the upregulation of TRIB3 was confirmed both in primary specimens from GBM patients and *in vitro* with GBM cell lines. Overexpression of specific TRIB3 transcripts promoted cell growth and migration *in vitro*, while knockdown of TRIB3 expression exerted a repressive effect on these cellular processes. The growth-promoting effect of TRIB3 was also demonstrated in a xenograft mouse model. Mechanistic studies further revealed that TRIB3 was able to suppress autophagic flux and that this suppression was responsible for TRIB3 silencing-induced proliferation and migration of GBM cells. These findings indicate that the suppression of autophagic flux by TRIB3 drives the invasion and proliferation of GBM cells, thus suggesting that TRIB3 is a potential novel therapeutic target for the treatment of glioma.

## INTRODUCTION

Glioma is an important condition affecting the nervous system and is the most invasive and malignant brain tumor in humans [1]. There is currently no treatment for glioma, and the average survival time for patients with glioblastoma (GBM) is less than eighteen months [2, 3]. However, because of its strong invasive ability and resistance to chemotherapy and radiotherapy, the clinical treatment of gliomas is still very difficult. Therefore, there is an urgent need for extensive research on the molecular mechanism of gliomas.

Tribbles 3 (TRIB3) is a pseudokinase and belongs to the Tribbles homologous family. TRIB3 has many kinase domains but does not contain 5'-adenosine triphosphate (ATP) binding and catalytic core motifs. Numerous studies suggest that TRIB3 is upregulated under various stresses, including oxidative stress, endoplasmic reticulum (ER) stress, and metabolic stresses [4]. Emerging studies have revealed that TRIB3 can be increased by various stimulations and regulate the signaling pathways of transforming growth factor-β, mitogen-activated protein kinase, and phosphatidylinositol 3-kinase (PI3K), thus playing an important role in glucose/lipid metabolism, cell differentiation, and cell survival [4]. Recently, several groups have found TRIB3 as a key factor regulating tumorigenesis and progression of tumors. In particular, TRIB3 is upregulated in various cancer tissues and tightly related to the poor prognosis of patients, including non-small-cell lung cancer [5], breast cancer [6], and colorectal cancer [7]. However, its exact roles in glioma remain unknown.

Autophagy is an evolutionarily conserved process in which cells engulf their proteins or organelles into vesicles and fuse with lysosomes to form autolysosomes, degrading the contents wrapped in them to achieve the metabolic needs of the cells themselves and to replenish certain organelles, so it is essential for cell growth regulation and internal interaction [8, 9]. In some cases, excessive autophagy can cause cell death [10–13]. The role of autophagy in cancer is very complicated. It has been reported that autophagy has both pro- and antitumor roles [14]. Autophagy can inhibit the progression of cancer progression by limiting tumor cell death and inflammatory reactions in the early stages of cancer [15, 16]. In contrast, autophagy is recognized as an accelerator in advanced cancer [17, 18]. Whether TRIB3 regulates the progression of glioma by modulating autophagy is still unknown.

In this study, combining *in vitro* and *in vivo* analyses, we investigated: i) the difference in the expression of TRIB3 in both primary specimens from glioma patients and GBM cell lines and that in controls; ii) the effects of overexpression and knockdown of TRIB3 on GBM cell progression *in vitro* and *in vivo*; and iii) the relationship between TRIB3 and autophagic flux in GBM. Our study indicates that the suppression of autophagic flux by TRIB3 drives the invasion and proliferation of GBM cells, and TRIB3 knockdown promotes autophagic flux and inhibits the malignant behavior of GBM cells. TRIB3 is a potential therapeutic target for the treatment of GBM.

## RESULTS

### TRIB3 is upregulated in human GBM

**Figure 1:**
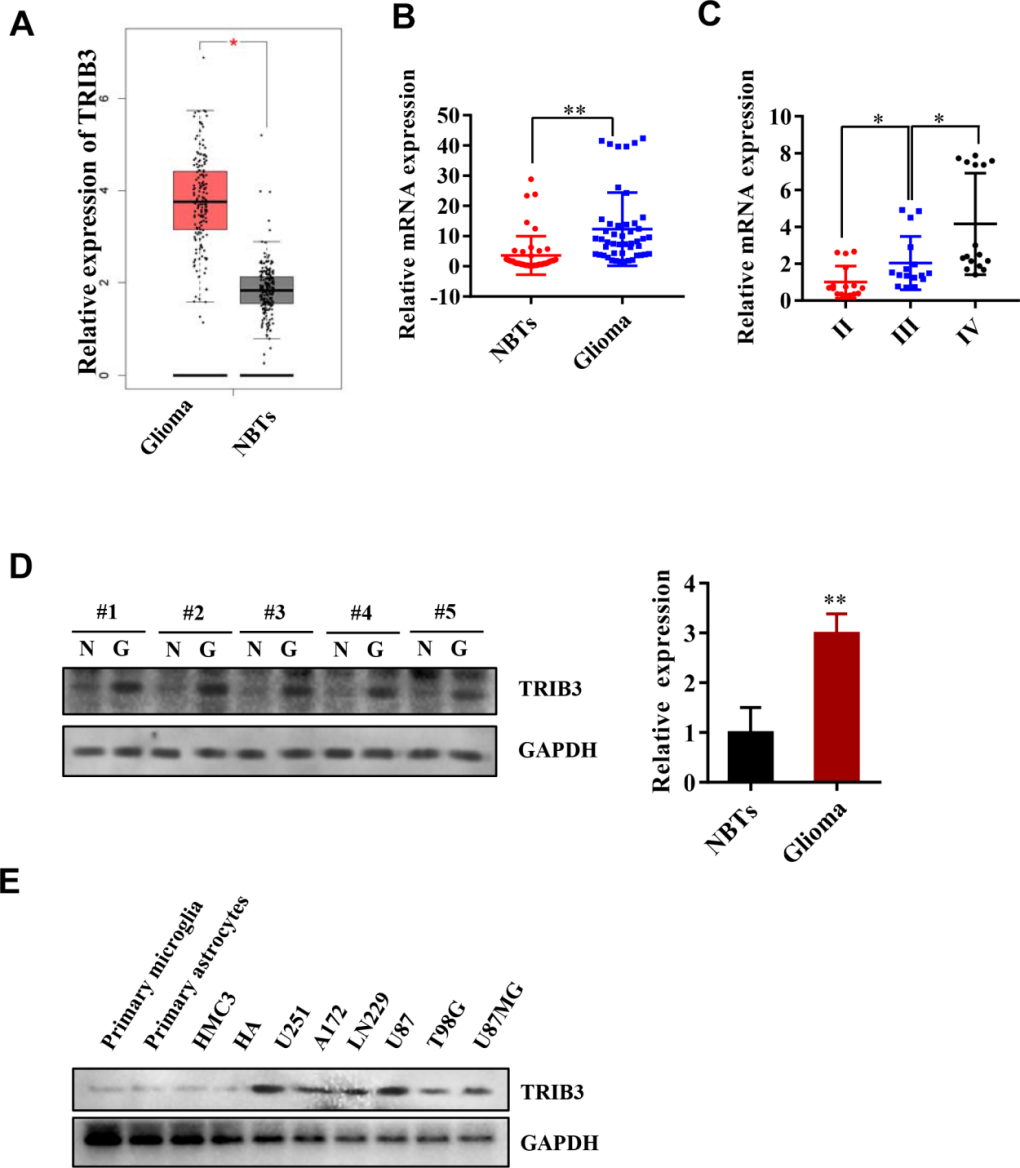
**TRIB3 is upregulated in human GBM.** (**A**) The expression level of TRIB3 from the TCGA database. (**B**) Q-PCR analysis of TRIB3 mRNA levels in glioma tissues (n = 48) and neighboring tissues (NBTs, n = 48). (**C**) Q-PCR analysis of TRIB3 mRNA levels in different grades of glioma. (**D**) The protein expression level of TRIB3 in glioma tissues and NBTs. (**E**) The protein expression level of TRIB3 in different normal brain cells and glioma cell lines. *p<0.05, **p< 0.01.

We began with analyzing the expression of TRIB3 in the Cancer Genome Atlas (TCGA) and found that the expression of TRIB3 in gliomas was increased compared with that in normal brain tissues (NBTs) (Figure 1A). To further validate the results found in the TCGA, we investigated the expression of TRIB3 in patient samples collected during brain surgery. Compared with that in NBTs, the mRNA level of TRIB3 was higher in glioma tissue (Figure 1B). We also divided the glioma tissue into three different grades according to the histopathological classifications formulated by WHO [19, 20] and found that the mRNA level of TRIB3 was related to the grade of glioma (Figure 1C). Then, we measured the expression of TRIB3 in GBMs and NBTs from several clinical samples and found that TRIB3 was increased in all GBM samples (Figure 1D). Next, we detected the expression of TRIB3 in glioma cell lines (U251, A172, LN229, U87, T98G, and U87MG) and the normal human cell line HMG3, HAs, primary microglial cells and primary astrocytes. TRIB3 was upregulated in glioma cell lines, especially in U87 and U251 cells (Figure 1E). These findings indicate that TRIB3 expression is increased in glioma.

### TRIB3 promotes the proliferation and migration of GBM cells

**Figure 2:**
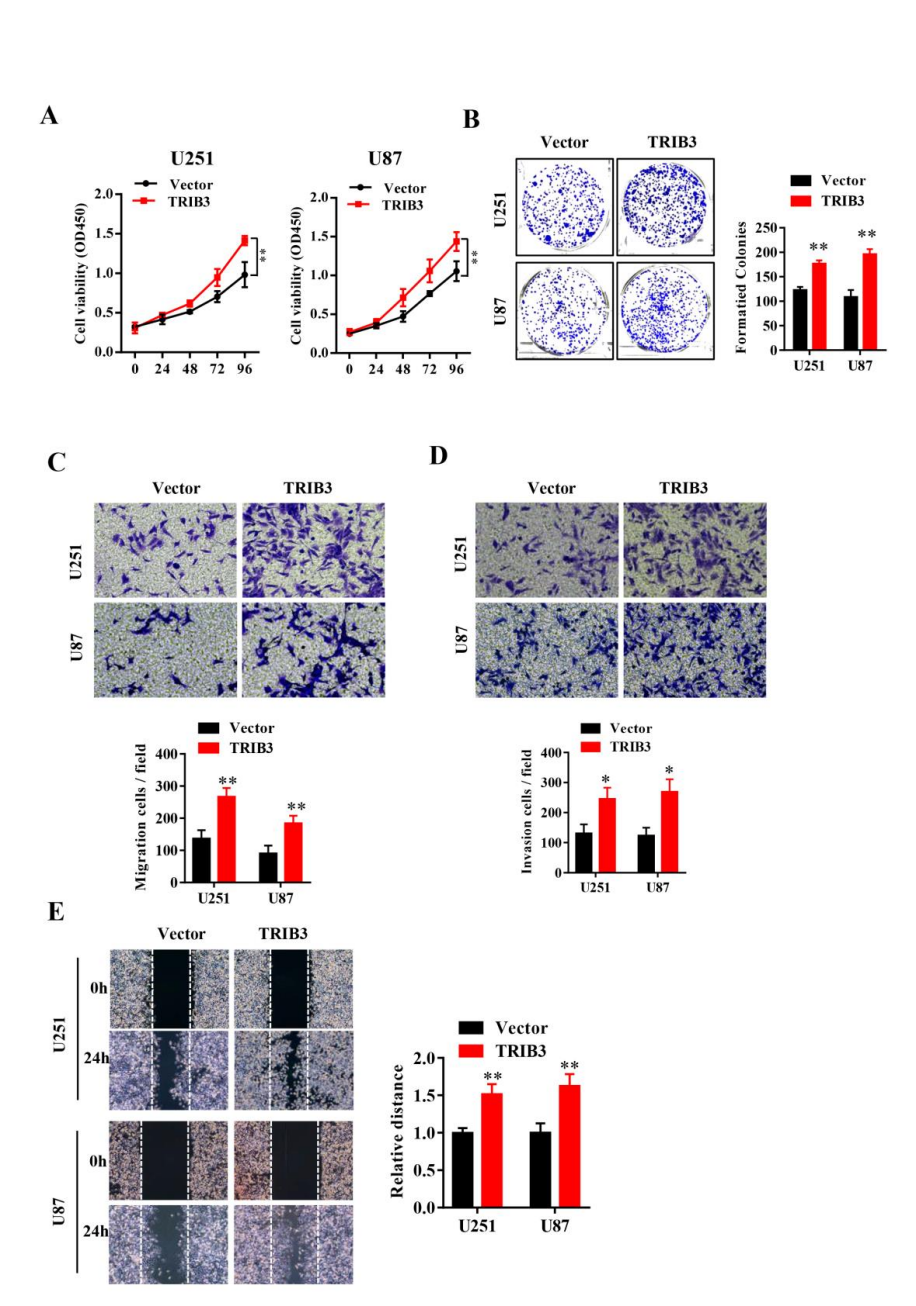
**TRIB3 overexpression promotes the proliferation and migration of GBM cells.** (**A**) The cell viability of U251 and U87 GBM cells was evaluated by CCK-8 assay. (**B**) Colony formation of U251 and U87 cells transfected with vector and TRIB3. (**C**, **D**) The cell migration and invasive ability of U251 and U87 cells were measured by Transwell assay. (**E**) The migration potential of U251 and U87 cells was evaluated by the wound healing test. *p<0.05, **p< 0.01.

To explore the function of TRIB3 in glioma, we transfected glioma cell lines with lentivirus carrying TRIB3 or vector, and the overexpression efficiency was verified by Western blot (data not shown). We assessed the cell viability after transfection at different times and found that GBM cells transfected with TRIB3 showed had higher cell viability than the vector control cells (Figure 2A). Next, we performed colony formation assays of GBM cells transfected with vector and TRIB3 and found that TRIB3 increased the colony formation of GBM cells. The cell migration and invasive ability of GBM cells were measured by Transwell assay and wound healing assay, and we found that upregulation of TRIB3 increased cell proliferation, migration, and invasion of GBM cells (Figure 2B–2E).

**Figure 3:**
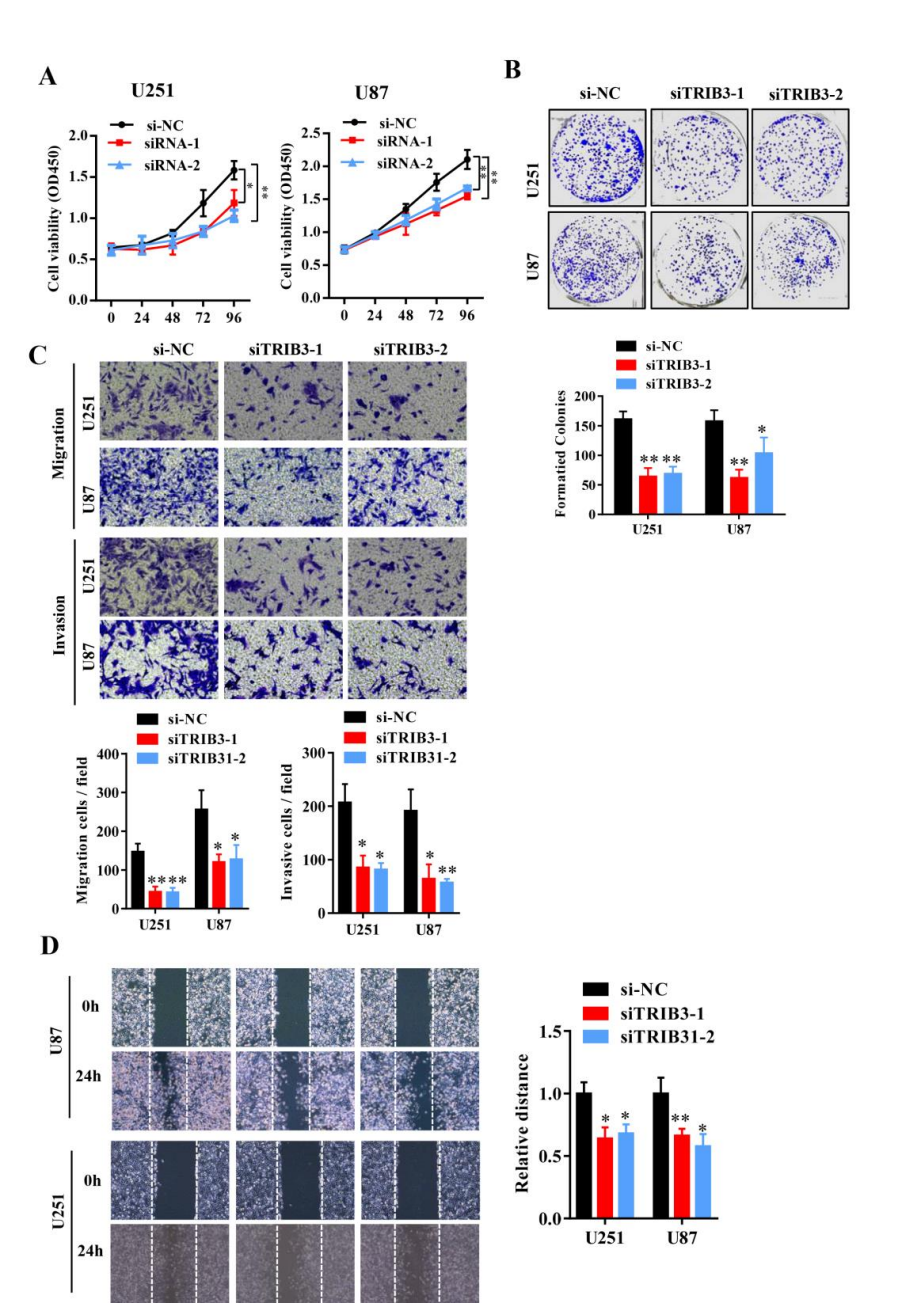
**Knockdown of TRIB3 impairs the proliferation and migration of GBM cells.** (**A**) The effect of TRIB3 knockdown on U251 and U87 cell proliferation was detected by CCK-8 assay at the indicated times. (**B**) The colony formation of U251 and U87 cells treated with si-NC and si-TRIB3. (**C**) Migration ability, and invasion ability were determined after the downregulation of TRIB3 in glioma cell lines using a migration assay and a Transwell assay. (**D**) The migration potential of U251 and U87 cells treated with si-NC and si-TRIB3 was evaluated by the wound healing test. *p<0.05, **p< 0.01.

To further explore the function of TRIB3 in glioma, we transfected glioma cell lines with a plasmid carrying shRNA targeting TRIB3 (sh-TRIB3-1 and sh-TRIB3-2), and the knockdown efficiency was verified by Western blot analysis (data not shown). We performed colony formation assays, Transwell assays and wound healing assays to determine the effects of TRIB3 downregulation on the colony formation, migration and invasion of GBM cells and found that TRIB3 downregulation decreased the viability, colony formation, migration and invasion of GBM cells (Figure 3A–3D). These results indicate that TRIB3 is involved in the process of cell proliferation, migration, and invasion of GBM cells.

### TRIB3 promotes glioma growth and metastasis *in vivo*

**Figure 4:**
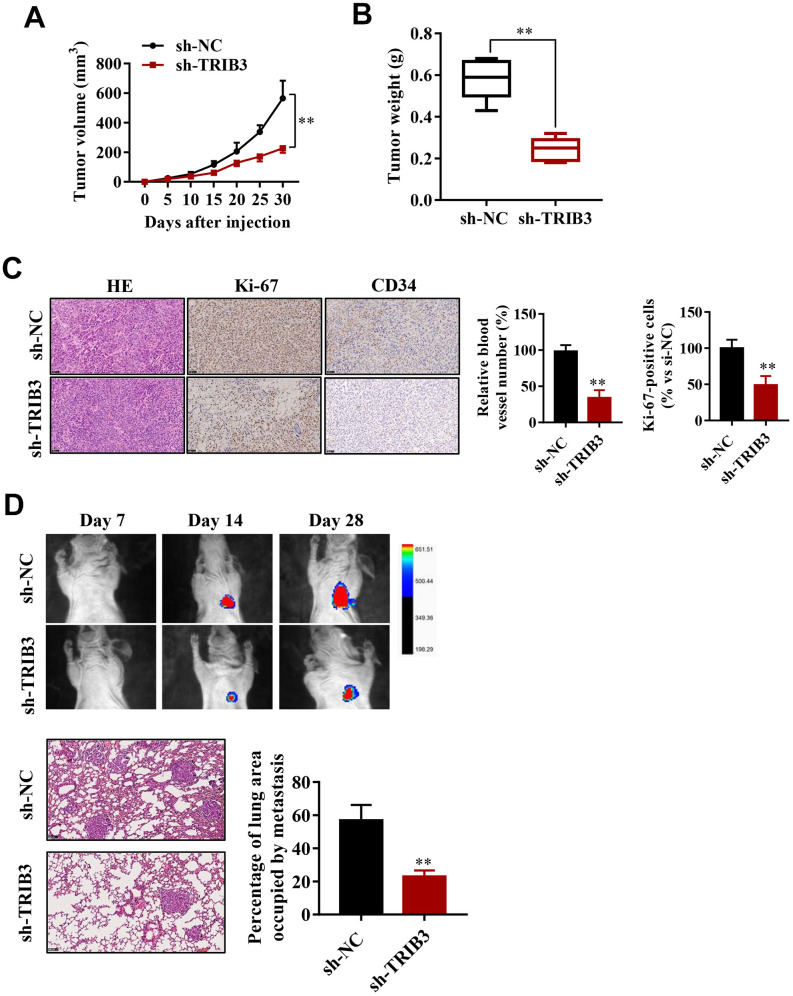
**TRIB3 promotes glioma growth and metastasis *in vivo*.** (**A**) Quantitative analysis of the tumor volume at the indicated times. (**B**) Quantitative analysis of the tumor weight. (**C**) HE staining and Ki-67 and CD34 immunohistological staining of tumors derived from scramble and sh-TRIB3 cells. (**D**) *In vivo* bioluminescent imaging of nude mice at days 7, 14, and 28 post-GBM cell implantation. Quantification of luminescence signal intensity on days 7, 14, and 28 after implanting the GBM cells. *p<0.05, **p< 0.01.

To explore the effect of TRIB3 inhibition on glioma growth, we used a xenograft mouse model. U251 cells stably transfected with sh-TRIB3 or empty vector were subcutaneously injected into nude mice. After four weeks, we found that sh-TRIB3 significantly reduced the tumor volume and tumor weight of sh-TRIB3 mice compared with the control group (Figure 4A, 4B). In addition, IHC analysis showed that there were fewer Ki-67-positive cells and CD34-positive cells in the si-TRIB3 group than in the control group, reflecting that si-TRIB3 decreased the number of proliferating cells and blood vessels (Figure 4C). These results indicate that silencing TRIB3 expression inhibits the growth of GBM cell-derived tumors in a mouse xenograft model.

Next, we evaluated whether TRIB3 influences the metastasis of GBM *in vivo*. U251 cells infected with lentivirus carrying sh-TRIB3 were injected into the tail vein of BALB/c nude mice. The luciferase signals were measured at days 7, 14, and 28 to monitor the growth of GBM tumor xenografts in the lung (Figure 4D). We found that the sh-TRIB3 group showed fewer luciferase signals at 14 and 28 days after injection. HE staining of the lungs of the different groups also showed that sh-TRIB3 inhibited the occupation of glioma in the lungs (Figure 4D). Altogether, we discovered that sh-TRIB3 effectively inhibited the metastasis and growth of U251 cells to the lungs of nude mice compared to those in the control group (Figure 4D).

### TRIB3 knockdown induces autophagy flux in GBM cells

**Figure 5:**
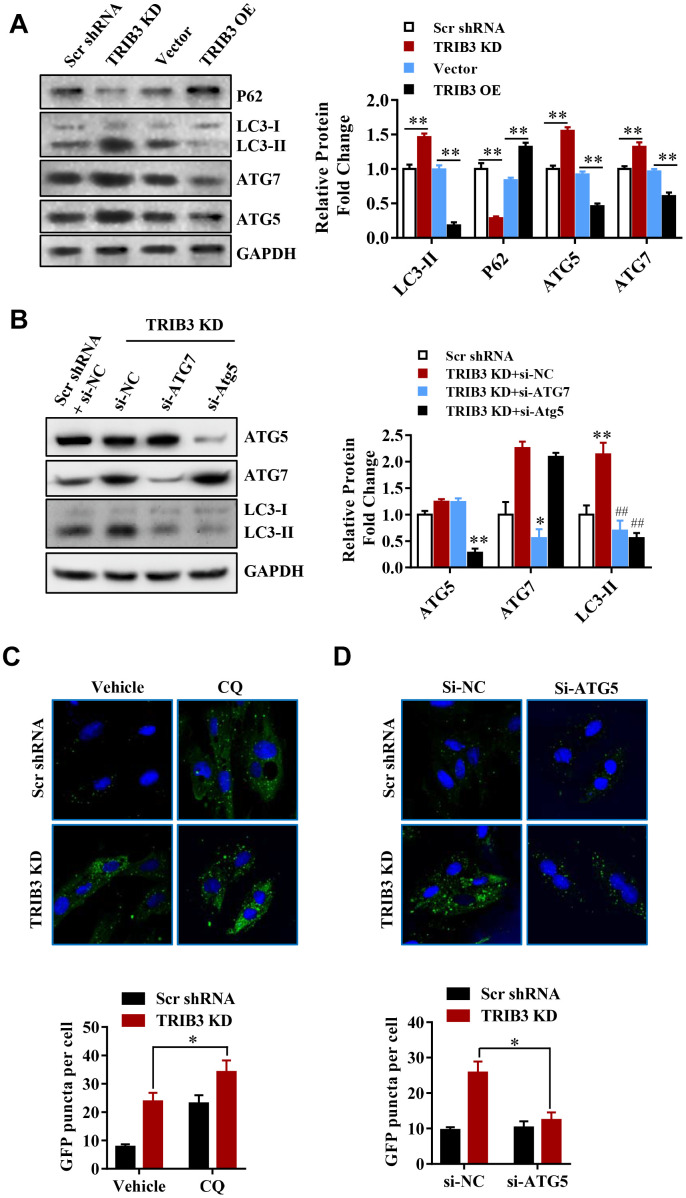
**TRIB3 knockdown induces autophagy flux in GBM cells.** (**A**) Western blot analysis to detect the protein levels of P62, LC3, ATG5, ATG7, and GAPDH (control for loading) in U251 cells treated with Ad-Scr, Ad-siTRIB3, Ad-vector, and Ad-TRIB3 for 48 h. Data are representative of 3 independent experiments. (**B**) Western blot analysis of LC3, ATG5, ATG7, and GAPDH in U251 cells treated with Ad-Scr+si-NC, Ad-siTRIB3+si-NC, Ad-siTRIB3+si-ATG7, and Ad-siTRIB3+si-ATG5 for 48 h. Data are representative of 3 independent experiments. (**C**) Fluorescence images of GFP-MAP1LC3B puncta in U251 cells pretreated with autophagy inhibitors (CQ) or vehicle (DMSO) followed by transfection of scramble RNA or TRIB3 siRNA for 48 h. (**D**) Fluorescence images of GFP-MAP1LC3B puncta in U251 cells transfected with si-NC or ATG5 siRNAs followed by transfection of scramble RNA or TRIB3 siRNA for 48 h. *p<0.05, **p< 0.01, ^##^p< 0.01 vs. TRIB3 KD + siNC.

Many reports have revealed that autophagy can facilitate cell death and inhibit tumor growth. To investigate whether TRIB3 knockdown regulates autophagy in GBM cells, we analyzed the expression of autophagy-related proteins by Western blot analysis. P62, a protein that is degraded through the autophagy process, decreased in TRIB3 knockdown cells (Figure 5A), while the level of LC3-II, a marker for autophagy, increased in TRIB3 knockdown cells. ATG5 and ATG7, key components of autophagic flux, were also increased in TRIB3 knockdown cells (Figure 5A). In contrast, when TRIB3 was overexpressed in GBM cells, the expression of p62 was increased and the levels of LC3, ATG5, and ATG7 were decreased compared to those in the control cells (Figure 5A).

These results further support the idea that TRIB3 knockdown enhances autophagy in GBM cells. Protein fingerprint analysis of the splits prepared from the treated cells showed that TRIB3 knockdown induced the formation of autophagosomes (Figure 5B). We transfected U251 cells with ATG5 or ATG7 siRNA to inhibit the protein expression of ATG5 or ATG7 in the presence of TRIB3 knockdown. Protein analysis showed that knocking out ATG5 or ATG7 significantly reduced the formation of LC3-II (Figure 5B). TRIB3 knockdown increased the number of GFP-MAP1LC3B dots, and U251 cells treated with CQ (3 μM) in the presence of TRIB3 knockdown still resulted in a cumulative increase in the number of GFP-MAP1LC3B dots (Figure 5C). In contrast, si-ATG reversed TRIB3 knockdown effects as described above. These data indicate that TRIB3 knockdown induces autophagy in GBM cells *in vitro*.

These results further support the idea that TRIB3 knockdown enhances autophagy in GBM cells. Next, we transfected GBM cells with ATG5 or ATG7 siRNAs in the presence of TRIB3 knockdown. We found that knockdown of ATG5 or ATG7 decreased LC3-II formation significantly in the presence of TRIB3 knockdown (Figure 5B). TRIB3 knockdown increased MAP1LC3B puncta, and treatment of U251 cells with CQ (3 μM) still led to increased accumulation of MAP1LC3B puncta even when TRIB3 was knocked down (Figure 5C). In contrast, si-ATG reversed the accumulation of MAP1LC3B puncta induced by TRIB3 knockdown. Altogether, the above data indicate that TRIB3 knockdown induces autophagy in GBM cells *in vitro*.

### TRIB3-mediated suppression of autophagy promotes the proliferation and migration of GBM cells

**Figure 6:**
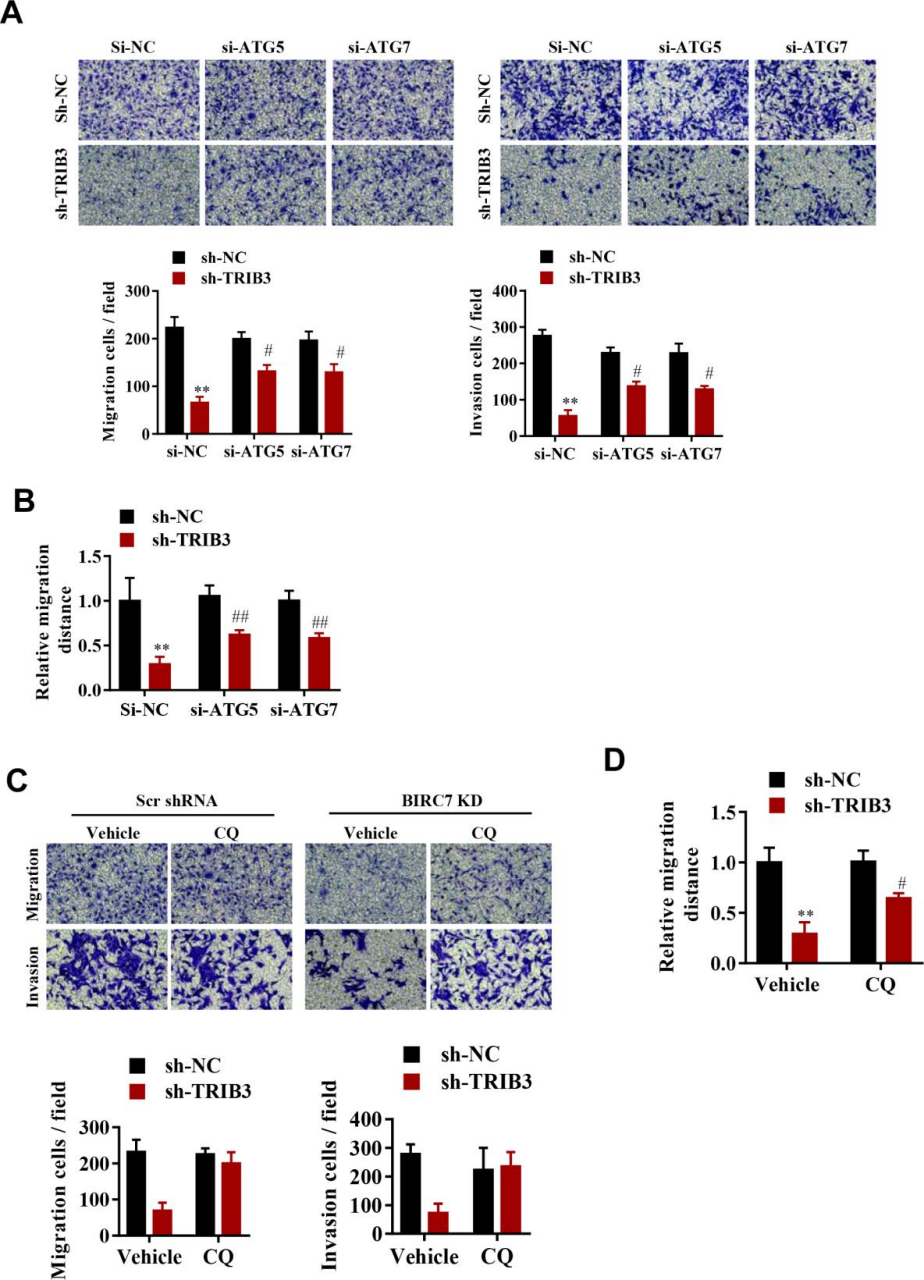
**TRIB3-mediated suppression of autophagy promotes the proliferation and migration of GBM cells. (A**, **B**) Migration ability (**A**) and invasion ability (**B**) were determined after the downregulation of TRIB3 coupled with downregulation of ATG5 or ATG7 in GBM cells using a migration assay and a Transwell assay. (**C**) Relative migration distance was determined in GBM cells using a migration assay. (**D**) Migration ability and invasion ability were determined after downregulation of TRIB3 and treatment with the autophagy inhibitor CQ or vehicle (DMSO) in GBM cells using a migration assay and a Transwell assay. E, Relative migration distance was determined in GBM cells using a migration assay. *p<0.05, **p< 0.01, ^#^p< 0.05 vs. TRIB3 KD + sh-NC.

To explore whether TRIB3-mediated suppression of autophagy promotes the proliferation and migration of GBM cells, cell migration and invasion were assessed after ATG5 or ATG7 knockdown. Interestingly, knockdown of both ATG5 and ATG7 significantly reversed the migration and invasion inhibition induced by TRIB3 knockdown (Figure 6A, 6B). As expected, inhibition of autophagy by CQ also significantly attenuated migration and invasion inhibition induced by TRIB3 knockdown in GBM cells (Figure 6C, 6D).

### TRIB3 deficiency enhances autophagy and impairs the malignant progression of GBM in mice

**Figure 7:**
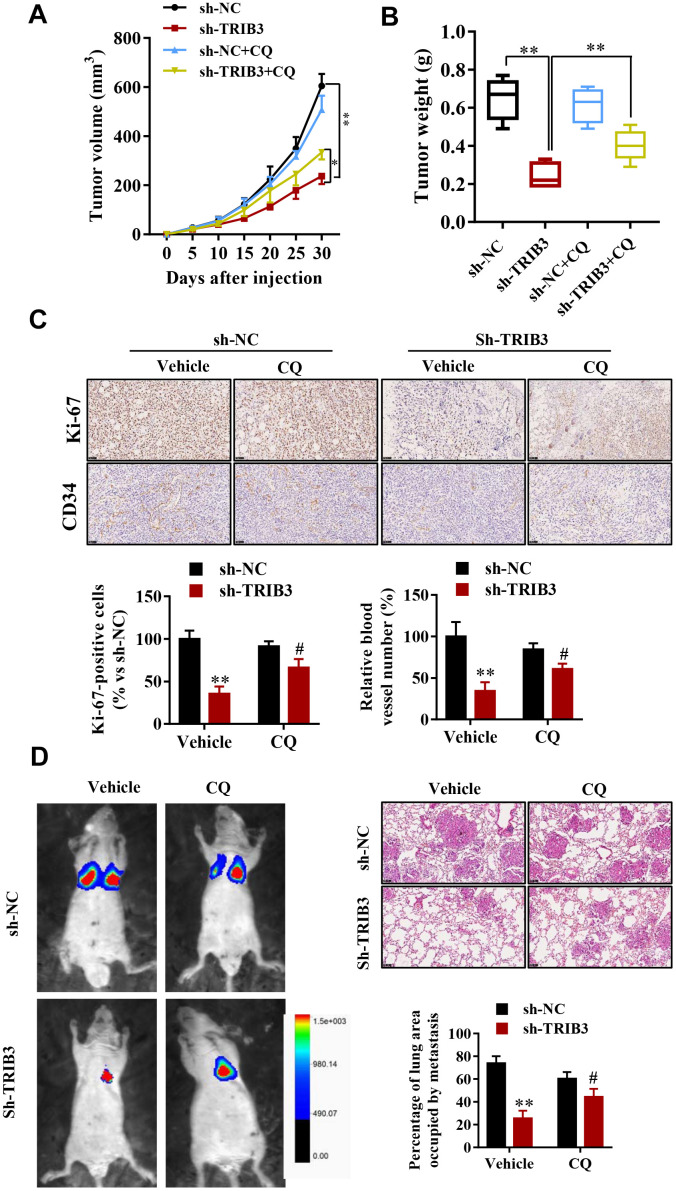
**TRIB3 deficiency enhances autophagy and impairs the malignant progression of GBM in mice.** (**A**, **B**) Tumor weight and volume in the sh-NC and sh-TRIB3 or sh-NC+CQ, sh-TRIB3+CQ groups. (**C**) Histological chemistry staining of Ki-67 and CD34 to measure the proliferation and angiogenesis of GBM cells in the sh-NC and sh-TRIB3 or sh-NC+CQ, sh-TRIB3+CQ groups. (**D**) Representative mice injected with modified sh-TRIB3- or sh-NC-expressing GBM cells. Downregulation of TRIB3 decreased lung metastases. *p<0.05, **p< 0.01, ^#^p< 0.05 vs. TRIB3 KD + sh-NC.

To determine the potential therapeutic efficacy of TRIB3 knockdown, tumor growth in response to treatment was investigated in a xenograft mouse model. U251 cells stably transfected with sh-TRIB3 or empty vector were injected into nude mice treated with CQ or not. After four weeks, we found that sh-TRIB3 significantly reduced the tumor volume and tumor weight of sh-TRIB3 mice compared with those of the control group, while these effects were abolished by CQ treatment (Figure 7A, 7B). In addition, CQ reversed the decreased numbers of Ki-67-positive cells induced by TRIB3 knockdown. In addition, CD34 staining showed that CQ also partially abolished the angiogenesis inhibition induced by TRIB3 knockdown (Figure 7C). Next, we evaluated whether the autophagy inhibitor CQ can inhibit the effects of TRIB3 knockdown on GBM metastasis *in vivo*. GBM cells infected with Lenti-sh-TRIB3 were injected into the tail vein of BALB/c nude mice with or without CQ, and luciferase signals were measured at day 28 to observe the growth of xenografts of GBM tumors in the lung (Figure 7D). Our results demonstrated that TRIB3 knockdown significantly reduced tumor metastasis *in vivo*. Significantly, the tumor metastasis effects of TRIB3 knockdown were partially reversed when autophagy was inhibited by CQ. Taken together, these data uncovered that the therapeutic effects of TRIB3 deficiency on GBM cells are dependent on autophagy flux and that TRIB3 deficiency enhances autophagy and impairs the malignant progression of GBM in mice.

## DISCUSSION

In this study, we demonstrate TRIB3 as a tumor promoter in glioma by combining *in vitro* and *in vivo* analyses. Bioinformatic analyses of public databases combining mRNA/protein expression of TRIB3 are upregulated in GBM patient samples and increase the poor prognosis of GBM patients, suggesting that TRIB3 may function as a tumor promoter and may have therapeutic value. Furthermore, overexpression of TRIB3 promotes cell growth and migration *in vitro*, while knockdown of TRIB3 in GBM cells decreases motility, proliferation, and clonogenic growth of GBM cells. The growth-promoting effects of TRIB3 on GBM cells were confirmed in a xenograft mouse model. Mechanistic studies further revealed that TRIB3 was able to suppress autophagic flux and that this suppression is responsible for TRIB3 silencing-induced proliferation and migration of GBM cells. Our study revealed that TRIB3 plays an important role in the promotion of glioma development and progression by suppressing autophagic flux, which drives the invasion and proliferation of GBM cells, suggesting that TRIB3 is a potential novel therapeutic target for the treatment of glioma.

The role of autophagy in cancer is still controversial. In one aspect, autophagy digests dysfunctional or damaged organelles through lysosomal degradation and reuses the products that are needed for cancer cells, such as GBM cells [21–23]. Therefore, autophagy is essential for maintaining homeostasis and mediating resistance to anticancer therapies such as radiation, chemotherapy, and some targeted therapies. Increasing evidence supports that treatment with autophagy inhibitors (such as CQ) enhances the effectiveness of many cancer treatments [24–26]. Li et al. revealed that caspase-mediated cleavage of Beclin1 inhibits autophagy and thus promotes apoptosis in human ovarian cancer cells [27]. In contrast, there is close crosstalk between cell apoptosis and autophagy [9, 11, 28]. Autophagy is important for the establishment of cell death in response to cancer therapy [29–31]. Treatment with temozolomide (used to treat some brain tumors such as glioma or anaplastic astrocytoma) has been shown to induce senescence in GBM cells and lead to a transient induction of autophagy to senescence [23, 32]. Other studies also found that autophagy transforms into senescence or apoptosis in the treatment of cancer cells [33, 34]. Our study revealed that TRIB3 plays a critical role in the promotion of glioma development and progression, but these protective effects were abolished by the autophagic flux inhibitor CQ *in vivo* and *in vitro*, indicating that autophagic flux inhibits the invasion and proliferation of GBM cells.

Several groups have demonstrated that TRIB3 plays a critical role in the regulation of tumorigenesis and tumor progression in recent years [35, 36]. However, its roles in glioma are still unknown. Much research has found a relationship between TRIB3 and P62, which is a key component of autophagic flux, and this TRIB3-P62 interaction abolishes the binding of LC3 and ubiquitinated substrates to P62, thus inducing the blockage of autophagic flux and subsequent defects in the clearance of ubiquitinated proteins [37]. By activating autophagic flux and the ubiquitin-proteasome system, inhibiting TRIB3 or interrupting the TRIB3-SQSTM1 interaction can attenuate melanoma growth and metastasis [36, 38]. We first found that autophagic flux mediates the antitumor effects of TRIB3 knockdown in GBM, but the molecular mechanism still needs to be further studied.

In summary, our findings indicate that the suppression of autophagic flux by TRIB3 drives the invasion and proliferation of GBM cells and that TRIB3 knockdown promotes autophagic flux and inhibits the malignant behavior of GBM cells. We predict that TRIB3 is a potential novel therapeutic target for the treatment of glioma that can be applied in the future.

## MATERIALS AND METHODS

### Cell lines and cell culture

GBM cell lines (U251, LN229, U87, T98G, A172, and U87MG), normal human HMG3 cells, HAs, primary microglial cells and primary glial cells were obtained from the Cell Bank of Type Culture Collection of the Chinese Academy of Sciences, Shanghai Institute of Cell Biology (Shanghai, China). The tumor cell lines were cultured in a 5% CO_2_ incubator at 37 °C in RPMI-1640 (Thermo, USA) containing 10% FBS, penicillin (100 U/mL) and streptomycin (100 U/mL).

### Patients and tissue specimens

A total of 48 glioma samples and paired normal samples were collected from the First Affiliated Hospital of Harbin Medical University. The samples were quickly frozen in liquid nitrogen and then stored at -80 °C until confirmed using qRT-PCR and Western blot analysis. The research ethics committee of the First Affiliated Hospital of Harbin Medical University approved the research in accordance with the Declaration of Helsinki.

### RNA isolation and RT-qPCR

qPCR was performed as described previously [39]. Briefly, tissue RNA was extracted from the glioma samples and corresponding normal tissue using TRIzol reagent (Thermo, USA). cDNA was generated by reverse-transcribed total RNA (1 μg) using oligo (dT) primers and ReverTra Ace reverse transcriptase (Toyobo). qPCR was performed using the ABI PRISM 7900 system (Applied Biosystems) with SYBR Green Real-time PCR MasterMix plus (TOYOBO) for quantification analysis. GAPDH was used for the housekeeping gene.

### Lentivirus vector construction and siRNA transfection

Lentivirus was the knockdown, and overexpression lentivirus vectors for TRIB3 were produced by OBiO (Shanghai, China). The siRNAs against TRIB3 (si-TRIB3), ATG5 (si-ATG5), and ATG7 (si-ATG7) and the negative control (NC) were synthesized by GenePharma (Shanghai, China). The siRNA sequence against TRIB3 is 5’-CTTCGTCCAGCCCCAGTCC-3’, the siRNA sequence against ATG5 is 5’- CTTGTTTCACGCTATATCA-3’, and the siRNA sequence against ATG7 is 5’- GGAGTCACAGCTCTTCCTT-3’. GBM cells were transfected with siRNAs with Lipofectamine 3000 Reagent (Thermo, USA) according to the manufacturer’s protocol.

### CCK-8 assay

Cell viability was evaluated by Cell Counting-Kit 8 (CCK-8) according to the manufacturer's protocol (MCE, China). siRNA or plasmid was transfected into GBM cells after 24 hours of implantation at a density of 2 × 10^3^ cells/well into a 96-well plate. After culturing for 2 days, the medium in each well was replaced with 100 μl of culture medium containing 10 μl of CCK-8 solution. The absorbance at 450 nm was measured by a spectrometer (Thermo Fisher, Rockford, IL, USA).

### Colony formation assay

GBM cells were plated into 12-well plates (1 × 10^3^ cells per well). The colonies were stained with 0.1% crystal violet (Sigma-Aldrich) after 2 weeks of culture and then washed with PBS twice. Visible violet colonies were counted and analyzed.

### Transwell assay

To evaluate the migration ability of GBM cells, 24-well Transwell chambers (Corning) with gelatin-coated polycarbonate membrane filters and Matrigel were used. The cells were suspended in FBS-free medium, and the transformed cell density was 5 × 10^4^/mL. Then, 100 μL of the lower chamber was added to 600 μL of medium containing 20% serum (HyClone, FBS). After 24 hours, the cells were stained with crystal violet (0.1%) for 15 minutes and then washed twice with PBS. ImageJ was used to observe and count the cells. The cells were observed with an optical microscope, and then different fields (6 fields per group) were randomly selected for counting.

### Wound healing assay

The cells were collected efficiently and then seeded into a 6-well plate (5 × 105/well). The cells were induced and cultivated to a confluence of nearly 90%. The GBM cells were scraped using a 200 μL pipette tip and cultured in FBS-free medium. Representative images were collected under the microscope 0 and 24 hours after the scratches were generated.

### *In vivo* tumor growth model

GBM cells transfected with sh-TRIB3 or interference control vector were harvested and suspended at a density of 1 × 10^8^ cells/ml in saline. As in previous studies, we injected 100 μL of cells subcutaneously into each mouse. We measured the volume of GBM tumors every 5 days and then sacrificed the mice and isolated and weighed the tumors after 30 days.

### Immunohistochemical (IHC) staining

After tumor tissue weighing, the tissues were embedded with OCT and frozen at -80°C. Six-micrometer-thick sections were made in a cryostat (Leica, Germany). The slides were fixed with 4% PFA, and 5% milk was used to block nonspecific binding sites of antibody at room temperature for 1 h. Slides were then incubated with anti-CD34 and anti-Ki-67 at room temperature for 2 h in a humid environment. The secondary antibodies were incubated for 2 h at room temperature, and then diaminobenzidine (DAB) was used as the chromogen. The brown areas within the nucleus or cytoplasm were considered Ki-67- and CD34-positive cells.

### Western blotting

Glioma and normal tissues were minced, homogenized, and digested in RIPA lysis buffer (with protease and phosphatase inhibitors, Thermo Scientific). Cells were scraped off culture plates on ice and lysed with RIPA (with protease and phosphatase inhibitors, Thermo Scientific). The resulting suspensions were centrifuged at 13,000 rpm for 20 minutes at 4°C, and the protein supernatant was collected. Protein samples were prepared for PAGE gel electrophoresis. Proteins were then transferred to a PVDF membrane (BioRad) for immunoblotting with relevant antibodies. The following antibodies were used in this study: TRIB3 (ab137526, Abcam), P62 (ab91526, Abcam), LC3 (ab51520, Abcam) and ATG5 (#15071, Cell Signaling), ATG7 (ab53255, Abcam) and GAPDH (#5174, Cell Signaling) served as loading controls.

### *In vivo* lung metastasis model

Four-week-old male BALB/c nude mice were used for the *in vivo* lung metastasis model. Different stable U251 cells were injected into the tail vein of mice (n= 5 each). The bioluminescence signal of lung metastasis from day 7 to day 28 was determined. Bioluminescent flux (photons/s/cm^2^/steradian) was used to assess lung metastasis. Metastatic progression was monitored and imaged using an IVIS-100 system (Caliper Life Sciences, MA, USA) 10 min after intraperitoneal injection of luciferin (300 mg/kg i.v.) in 80 μl of saline. After 4 weeks, all mice were sacrificed, and then immunohistochemical analysis and hematoxylin/eosin (H&E) staining were performed.

### Statistical analysis

Data are presented as the mean ± SEM. Statistical analyses were performed with GraphPad Prism software (version 8.1). Statistical significance was assessed via unpaired Student's t-test or one-way analysis of variance (ANOVA) followed by Bonferroni's multiple testing correction as appropriate. Differences were considered statistically significant at the level of P<0.05.

### Ethics statement

All animal procedures were approved by the Institutional Animal Care and Use Committee (IACUC) of Harbin Medical University.
